# Canine Idiopathic Epilepsy as a Natural Animal Model for Human Epilepsy: A Scoping Review Highlighting Metabolic Perspectives Beyond the Brain

**DOI:** 10.3390/nu18111734

**Published:** 2026-05-28

**Authors:** Giulia Cabri, Sofie F. M. Bhatti, Lieselot Y. Hemeryck, Paul Boon, Holger A. Volk, Myriam Hesta, Fien Verdoodt

**Affiliations:** 1 Equine and Companion Animal Nutrition, Department of Morphology, Imaging, Orthopedics, Rehabilitation and Nutrition, Faculty of Veterinary Medicine, Ghent University, Salisburylaan 133, 9820 Merelbeke-Melle, Belgium; giulia.cabri@outlook.com (G.C.); fien.verdoodt@ugent.be (F.V.); 2 Small Animal Department, Faculty of Veterinary Medicine, Ghent University, Salisburylaan 133, 9820 Merelbeke-Melle, Belgium; sofie.bhatti@ugent.be; 3 Laboratory of Integrative Metabolomics (LIMET), Department of Translational Physiology, Infectiology and Public Health, Faculty of Veterinary Medicine, Ghent University, Salisburylaan 133, 9820 Merelbeke-Melle, Belgium; lieseloty.hemeryck@ugent.be; 4 Department of Neurology, Ghent University Hospital and 4Brain, Ghent University, C. Heymanslaan 10, 9000 Ghent, Belgium; paul.boon@uzgent.be; 5 Department of Small Animal Medicine and Surgery, University of Veterinary Medicine Hannover, 30559 Hannover, Germany; holger.volk@tiho-hannover.de

**Keywords:** epilepsy, dog, human, preclinical, metabolome, animal model

## Abstract

**Background:** Emerging evidence indicates that epilepsy extends beyond the brain, involving systemic metabolic, immune, and microbiome perturbations that shape neuronal excitability and treatment response. Canine idiopathic epilepsy (CE) offers a naturally occurring model with strong electrophysiological, pharmacological, and clinical homology to human epilepsies. **Methods:** This scoping review was conducted according to the PRISMA-ScR guidelines. A systematic literature search was performed in Web of Science and MEDLINE (PubMed) to identify original studies reporting metabolic, immunometabolic, or neurochemical alterations in CE compared with healthy controls. Eligible studies included peer-reviewed original research involving client-owned dogs diagnosed with CE according to international consensus criteria (IVETF guidelines). Studies focusing exclusively on genetics or neuroimaging without metabolic outcomes were excluded. Titles, abstracts, and full texts were screened for eligibility, and data were extracted from included studies using a standardized approach. Identified metabolic domains were synthesized narratively and grouped into functional systems, including amino acid and lipid metabolism, micronutrients, neurotransmission, oxidative stress, inflammation and immunology, endocannabinoid signalling, microRNAs, and gut–brain axis-related pathways. In a second step, the identified metabolic domains were evaluated for translational relevance through a targeted, non-systematic narrative synthesis of the human epilepsy literature. This approach aimed to assess cross-species parallels and to provide a conceptual framework to guide future research, rather than to perform a comprehensive systematic review of metabolic alterations in human epilepsy. **Results:** Across CE studies, consistent alterations were observed in multiple interconnected functional systems, including metabolic, immune, and gut–brain axis pathways, in agreement with findings reported for human epilepsy. These data support a model of epileptogenesis involving systemic dysfunction beyond the central nervous system. Translationally, these findings suggest opportunities for biomarker development, patient stratification, and mechanism-based interventions, including dietary and metabolic approaches (e.g., medium-chain triglyceride supplementation), microbiome modulation, and immunometabolic targeting. The current evidence is limited by small and heterogeneous cohorts, potential confounding effects of antiseizure medications, variability in dietary and fasting conditions, breed-related effects, and a predominance of associative over causal relationships. **Conclusions:** This review positions CE as a reference framework for future research into epilepsy metabolism, integrating current evidence and its translational relevance to human disease. The findings support a shift toward a systems-level view of epileptogenesis, involving interconnected metabolic, immune, and gut–brain axis pathways beyond the brain. CE represents a valuable translational model to identify shared mechanisms, inform biomarker discovery, and guide the development of mechanism-based therapeutic strategies across veterinary and human epilepsy.

## 1. Introduction

Epilepsy is one of the most common chronic neurological disorders in humans, highly affecting the quality of life of patients and their families [1]. While epilepsy affects around 50 million people worldwide [2], the underlying etiology remains unclear in 32% of cases [3]. Additionally, the currently available antiseizure medications (ASMs) remain unable to fully control epileptic seizures in approximately one third of people with epilepsy [4]. These numbers stress the importance of further research to better characterize the pathophysiology of epilepsy and its management. Recent evidence supports a more integrative perspective on epilepsy [5,6,7,8], recognizing that its pathophysiological manifestations extend beyond the central nervous system (CNS) and are also reflected in systemic metabolic alterations [9]. Characterization of these metabolic pathways, systems, and states may help to further elucidate the underlying etiologies, inform the development of novel therapeutic strategies, including nutritional interventions, and ultimately improve the clinical management of epilepsy.

Human epilepsy encompasses a heterogeneous group of disorders characterized by diverse etiologies and complex pathophysiological mechanisms [10]. The variability in clinical presentation and underlying causes among individuals underscores the need for a tailored therapeutic approach considering the specific patient and disease characteristics, evolving towards personalized medicine [11]. Emerging technologies such as metabolomics, especially when integrated with established disease models, offer promising avenues to advance diagnostic and therapeutic options in epilepsy [9].

Canine idiopathic epilepsy (CE) is an umbrella-term encompassing genetic epilepsy, suspected genetic epilepsy, and epilepsy of unknown cause [12]. Its diagnosis is based on internationally recognized criteria to exclude structural or reactive causes resulting in epileptic seizures [13]. As such, CE represents a robust, naturally occurring model for human epilepsy types of genetic or unknown origin. Its translational value is supported by converging electrophysiological, pharmacological, and clinical evidence [14,15,16,17]. Notably, for example, dietary supplementation with medium-chain triglycerides has demonstrated efficacy across species, including rodents, dogs, and humans [18]. Simultaneously, the intestinal canine microbiome resembles the human microbiome better than that of rodents [19], reinforcing the relevance of the canine model to study potential pathways and systems of interest, like the gut–brain axis. However, direct cross-species comparisons of metabolic profiles remain limited, despite growing interest in molecular endotyping, i.e., the identification of distinct biological subtypes, to refine epilepsy classification and treatment strategies [20].

The dog offers several advantages as a translational model: (1) spontaneous seizure occurrence eliminates the need for artificial induction; (2) clinical manifestations closely resemble those observed in human epilepsy; (3) shared environmental exposures with humans enhance ecological validity; and (4) dietary intake can be standardized using fixed-formulation, nutritionally complete diets, an approach that is less feasible in human patients due to dietary heterogeneity and variable compliance. These latter factors (3 and 4) are particularly critical, as environmental and dietary variables are recognized as major confounders in human clinical research [21]. Importantly, the use of naturally affected dogs creates reciprocal benefits: insights translate to human epilepsy and simultaneously improve clinical outcomes in veterinary practice. This dual-impact, real-world model complements and extends traditional experimental approaches.

By delineating shared mechanisms and species-specific features, we aim to clarify the translational relevance of the canine model and provide a comprehensive overview of the existing knowledge in CE. The objective of this review was (1) to systematically identify the original studies in CE comparing metabolic pathways, systems, and states to a control group of healthy dogs; and (2) to explore the relevance of these identified pathways, systems, and states for human epilepsy. To reach this objective, a broad field of evidence must be mapped, and diverse study types will need to be integrated. Consequently, a scoping review provides the most suitable framework for this work [22]. This framework lays the groundwork for future research into metabolic perspectives beyond the brain, thereby ultimately facilitating potential advancements in diagnostic and therapeutic strategies for both veterinary and human patients.

## 2. Methods and Search Criteria

This study was conducted as a scoping review to systematically evaluate current evidence concerning the metabolic pathways, systems, and states implicated in CE, while a complementary narrative review underscores the convergence and divergence of these findings with the human literature. The methodology adheres to the PRISMA-ScR (Preferred Reporting Items for Systematic Reviews and Meta-Analyses extension for Scoping Reviews) reporting guidelines, emphasizing methodological rigour when identifying key concepts and research gaps in the field (Supplementary File S1).

**Figure 1:**
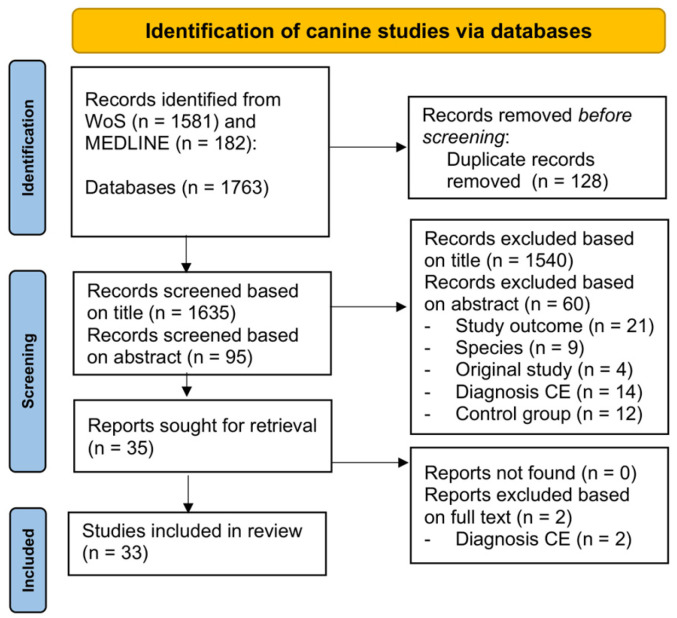
PRISMA flowchart showing the applied literature search strategy for canine epilepsy research (first step). Adapted from Page MJ et al. BMJ 2021;372:n71. doi: 10.1136/bmj.n71 [23] (CC BY 4.0).

In the first step, a literature review was conducted to identify studies investigating metabolic pathways in CE, following the PRISMA-ScR guidelines (Figure 1, Supplementary Files S1 and S2). The research question guiding this step was: “In dogs with CE (P), can we identify original studies comparing aspects of the metabolism (I) to a control group of healthy dogs (C), to detect alterations in specific metabolites or metabolic pathways (O)?”. The scoping search was conducted on 22 August 2025 using the Web of Science (WoS) and MEDLINE (via PubMed) databases. For WoS, the search string “(dog OR canine) AND (epileps*) (All Fields)”, followed by a filter to exclude “document types: review article”, was applied. In MEDLINE, the search string: “Dog OR canine,” AND “Epilep*,” was applied, with a publication type filter to include clinical trials, comparative studies, multicentre studies, evaluation studies and observational studies. After removing duplicates, the unique titles and abstracts were independently screened by the first and last author. Inclusion criteria were: (1) original research articles; (2) studies involving dogs diagnosed with idiopathic epilepsy in accordance with the international guidelines, i.e., CE [13]; and (3) studies including healthy dogs as a control group. Studies focusing solely on genotyping or medical imaging techniques were excluded. Both authors then evaluated the full text against the inclusion criteria, and studies were additionally excluded if one of the inclusion criteria was not met. The full list of screened records is available in Supplementary File S2. Finally, data were manually extracted by the first and last author from the result sections of the included articles. A critical appraisal of the individual CE studies was performed using the Levels of Evidence hierarchy (see: https://legacyfileshare.elsevier.com/promis_misc/YJPSU-Levels-of-Evidence.pdf (accessed 5 May 2026)), and the results are summarized in Table 1.

In the second step, metabolic pathways, systems and/or states of interest identified in the canine literature were further examined for their relevance to human epilepsy. For each of these, a narrative synthesis of the human literature complemented the CE data to determine whether similar alterations have been reported in human epilepsy studies. The identified metabolic pathways, systems and/or states will be discussed in this manuscript, organized according to the affected functional aspects.

It is important to note that no systematic search strategy was applied specifically to the human literature. Consequently, a selection bias toward the metabolic pathways previously identified in CE is inherent in this work. However, the primary objective of this study was to serve as a reference for future researchers by summarizing the current knowledge in CE and providing insights into how these findings relate to human epilepsy. A comprehensive systematic description of metabolic alterations in human epilepsy was deemed beyond the scope of the present work.

## 3. Results

The literature retrieved on CE through our scoping search strategy yielded 33 publications (summarized in Table 1), encompassing analyses of cerebrospinal fluid (CSF), brain tissue, blood, and fecal samples. All studies were conducted on client-owned dogs affected by CE in comparison with a healthy control group. Some studies additionally included extra control groups, like dogs with structural epilepsy or meningoencephalitis of unknown origin. Several studies further stratified the CE population into subcategories, such as ‘controlled’ or ‘mild phenotype’ versus ‘uncontrolled’ or ‘drug-resistant,’ and ‘treated’ versus ‘untreated.’ The number of dogs with CE enrolled in individual studies ranged from 4 to 92, while healthy control groups included 4 to 127 dogs. Across these investigations, specific metabolic and physiological mechanisms were identified, many of which appear interrelated and intricately involved in the pathogenesis of CE, with potential relevance to human epilepsy.

The data and results retrieved in this review are presented as different sections based on the functional categories of metabolic alterations described in CE: inflammatory and immune pathways, microbiota–gut–brain axis, oxidative stress, lipid metabolism, amino acid and protein metabolism, minerals, trace elements and vitamins, neurotransmission, endocannabinoid system, and microRNA. In each section, we first summarize the existing research on CE and subsequently compare these findings with evidence from studies on people with epilepsy (PWE), with the aim of delineating cross-species similarities and divergences.

## 4. Inflammatory and Immune Pathways

### 4.1. Neuroinflammation

#### 4.1.1. Canine Epilepsy

Although CE is traditionally considered a non-inflammatory epilepsy subtype [13], emerging evidence suggests a more nuanced pathophysiological profile, with each epileptic seizure inducing secondarily inflammatory processes [57]. Regarding epilepsy neuroinflammation, three canine studies were identified. One study revealed increased Heat Shock Protein 70 (HSP70) expression, a key damage-associated molecular pattern (DAMP), in the piriform lobe in CE [29]. Second, the CSF concentration of TNF-α, a pro-inflammatory cytokine, was increased in CE versus healthy dogs [32]. Third, serum creatine kinase (CK), a biomarker for tissue damage, was shown to be increased in CE [31]. Notably, this increase was primarily caused by an increase in the isoenzyme predominantly found in the brain, i.e., CK-BB, thus revealing neuron damage and leakage via the BBB. However, CE could not be differentiated from other CNS diseases, like inflammatory or degenerative disorders based on CK [31].

#### 4.1.2. Human Epilepsy

Similarly to dogs, human research highlighted an important role for neuroinflammation within the pathophysiology of epilepsy [58,59], for which similarities and divergences described in the literature are depicted in Table 2. Similar to CE, increased HSP70 levels have been identified in the CNS of PWE. One study found overexpressed HSP70 in epileptic vs. non-epileptic brain tissue from PWE with a focal drug-resistant epilepsy type [60], while another study identified increased HSP70 immunoreactivity in the surgically removed hippocampi of PWE with mesial temporal lobe epilepsy [61]. Additionally, the CSF of PWE following status epilepticus showed significantly higher HSP70 levels compared to either controls or PWE experiencing epileptic seizures not including status epilepticus [62]. Moreover, increased CSF pro-inflammatory cytokines, including TNF-α, like in CE, have been detected in multiple types of PWE [58,63,64].

#### 4.1.3. General Remarks on Neuroinflammation

Neuroinflammation has indeed long been recognized as a contributing factor in epilepsy, first as a consequence of recurrent seizures, perpetuating a self-reinforcing inflammatory cycle [65]. Additionally, evidence from experimental animal models indicated that neuroinflammation also precedes seizure onset, implicating it as a potential initiator of epileptogenesis [66]. In the brain, microglia activation results in the release of DAMPs [67], cytokines [68] and effector pathways, like COX-2 [69]. Among the DAMPs implicated in epilepsy, High-Mobility Group Box 1 (HMGB1) and HSP70 have emerged as key mediators in experimental rodent models and PWE [70]. Moreover, the blood–brain barrier (BBB) function is hampered in PWE [71] and CE [72], causing peripheral immune cells and molecules to enter the brain. The literature on CE seems to support this theory of a self-perpetuating inflammatory cycle; however, no causation has been studied yet. The current studies only reveal correlations between inflammatory markers and CE, while ASM management was not described nor included as confounder, which is considered an important limitation.

### 4.2. Peripheral Inflammation

As highlighted by the primary aim of this review, disturbances in epilepsy extend beyond the CNS, and this is equally true for inflammatory processes [73]. Accordingly, peripheral inflammatory markers may offer complementary insights. Similar to neuroinflammatory processes, peripheral inflammation typically begins with the recruitment of inflammatory cells and the release of pro-inflammatory cytokines, which in turn drive the synthesis of acute-phase proteins (APP) such as C-reactive protein (CRP), haptoglobin, and ceruloplasmin [74]. To avoid redundancy, APP other than CRP will be addressed under ‘protein metabolism’. The release of APP is ultimately followed by metabolic alterations, which is hypothesized to further reinforce the self-perpetuating inflammatory cycle.

#### 4.2.1. Canine Epilepsy

The inflammatory hematological cells in CE are characterized by a significant greater neutrophil to lymphocyte ratio [27], revealing primarily neutrophil-mediated inflammation. Additionally, increased DAMPs, i.e., serum HMGB1 [28], and cytokines, serum IL-1β [26], have been identified in CE. Regardless of epilepsy etiology, IL-1β was increased, showing no differences between idiopathic and structural epilepsy [26]. While serum CRP did not significantly increase in CE versus healthy dogs [30], elevated serum CRP levels were detected in dogs with structural epilepsy, suggesting seizure-induced inflammation may differ between epilepsy subtypes [27,75]. However, seizure severity in CE had an influence, with dogs experiencing cluster seizures showing higher serum CRP levels than dogs without clusters [27].

**Figure 2:**
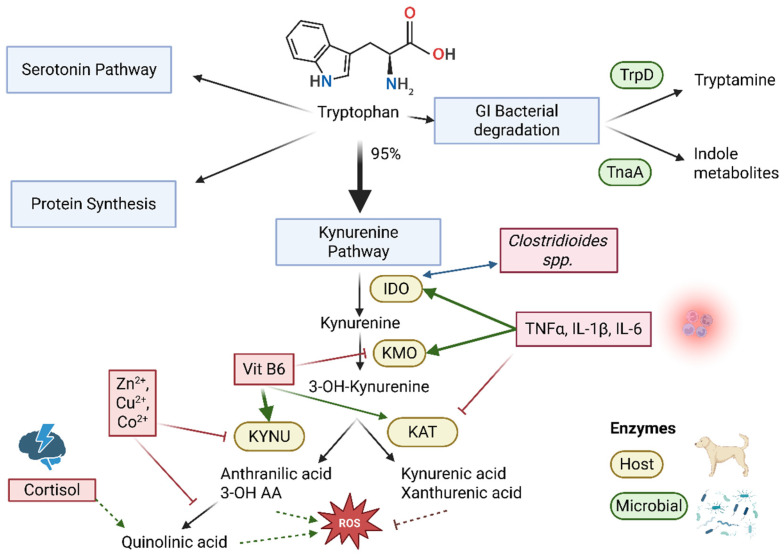
Schematic overview of the primary host and gastrointestinal microbial tryptophan metabolism, with a focus on the kynurenine pathway, and its interactions with inflammation, (oxidative) stress, gastrointestinal microbiota, vitamins and minerals, based on the previous literature [77,78,79]. TrpD: Tryptophan decarboxylase; TnaA: Tryptophanase; IDO: Indoleamine 2,3-dioxygenase; TNF: Tumour Necrosis Factor; IL: Interleukin; KMO: Kynurenine 3-monooxygenase; OH: hydroxy; Vit: vitamin; KAT: Kynurenine aminotransferase; Kynu: Kynurenine hydrolase; 3-OH AA: 3-hydroxy anthranilic acid. Created in BioRender.com.

Metabolic studies further support an inflammatory component in CE by identifying elevated plasma lipids, i.e., oleoylethanolamide (OEA), a PPAR agonist with anti-inflammatory properties, and 11,12-DHET [45]. For the latter, bioactivity is not well documented, while 11,12 epoxyeicosatrienoic acid (11,12 EET), a substrate for 11,12-DHET, has been shown to suppress seizures in mouse hippocampus [76]. Furthermore, drug-resistant CE was characterized by elevated plasma xanthurenic acid and reduced vitamin B6 levels [38]. These suggest inflammatory activation of the kynurenine pathway, which is recognized as the major metabolic pathway for tryptophan [77,78,79], and is schematically displayed in Figure 2. Additionally, 4-guanidinobutanoic acid, a proinflammatory substrate for the blood–brain barrier creatine transporter [80,81], was increased in drug-resistant CE [38]. Fecal metabolomics of the same study population revealed increased histamine in drug-resistant CE, together with higher serotonin in mild CE [41], indicating altered tryptophan metabolism and intestinal inflammation potentially linked with the severity of disease [82,83].

#### 4.2.2. Human Epilepsy

Starting with the inflammatory hematological cell types, similar to CE, higher neutrophil to lymphocyte ratios in PWE were indicated by recent meta-analyses [84,85]. Moreover, this ratio was significantly elevated in PWE with same-day seizure recurrence [86]. However, the relationship between epileptic seizures and neutrophil to lymphocyte ratio does not appear linear [87].

At the cytokine level, like in CE, increased IL-1β and TNFA-α were detected in the serum of PWE [88]. In addition, significantly higher blood CRP levels were found in adults with epilepsy, but not children [89]. In contrast, a study in 2022 did find significantly higher serum CRP levels in childhood epilepsy, and decreased CRP following treatment with levetiracetam [90].

The specific plasma lipids detected in CE, i.e., 11,12 DHET and OEA, have not yet been identified in PWE. Conversely, xanthurenic acid, a kynurenine pathway metabolite, was also increased in children with epileptic spasm syndrome [91], with a significant increase in children non-responsive to adrenocorticotropic hormone [92]. These findings suggest a link between epilepsy, the kynurenine and corticoid stress metabolism in both species.

#### 4.2.3. General Remarks on Peripheral Inflammation

The CE literature, similar to the literature on PWE, indicates a correlation with inflammation. For CE, this is based on four studies with a level of evidence of III, and one level II study. However, no causal links have been examined, leaving open the question as to whether inflammation is a cause, consequence, or both. Additionally, details of ASM management were included in only two of the CE studies, while its influence remains inconclusive. The potential influence of ASM on inflammation in PWE is considered mixed and ASM-specific [93,94,95], warranting further investigation. Rodent models showed that the efficacy of ASM was hampered with induced inflammation in one study [38,96], while chronic levetiracetam administration stimulated xanthurenic acid production by brain cells in another study [97]. In the canine study detecting increased plasma xanthurenic acid, it was increased only in drug-resistant CE, of which only 7/27 received levetiracetam [38]. This suggests that levetiracetam administration is unlikely to account for the observed increase. Conversely, valproic acid has been shown to reduce inflammatory microglia overaction via histone deacetylase inhibition in vitro [98] and, together with carbamazepine, reduce the levels of proinflammatory cytokines produced by human peripheral immune cells in vitro [99]. To the best of our knowledge, these anti-inflammatory effects have not been corroborated in a clinical setting. The specific interaction between ASM and inflammations remains a significant research gap in both CE and PWE.

### 4.3. Innate Immune System

#### 4.3.1. Canine Epilepsy

The complement cascade, a key interface between innate immunity and inflammation, revealed significantly increased serum C3 and C4 levels in CE, regardless of ASM status or seizure timing [24]. Notably, dogs with a mean seizure frequency ≥ 3/month exhibited higher C3 levels, implicating complement activation in disease severity [24].

#### 4.3.2. Human Epilepsy

The blood complement cascade was shown to be involved in the discrimination of PWE vs. controls [100], as well as in children suffering from febrile seizures [101]. In adults with idiopathic generalized epilepsy specifically, decreased C3 and C4 serum levels were detected [102]. More recently, lower complement components were detected in patients with DR epilepsy, together with a sex-dependent effect [103]. These findings are contrasting the increased C3 levels detected in CE [24], potentially revealing a species-specific complement response.

### 4.4. Adaptive Immune System

#### 4.4.1. Canine Epilepsy

In CE, one study reported significantly higher serum and CSF IL-17 concentrations, elevated stimulated Th17 cell counts, and a slight positive correlation between Th17 cell count and seizure severity [34]. Another study on CE reported undetectable concentrations of D-dimers in the CSF [25], while more recently, hyperfibrinolysis in CE treated with phenobarbital [104] was detected. One canine case of epilepsy caused by an autoimmune encephalitis has been described [105]. However, a prospective study evaluating paired CSF and serum samples could not detect neuronal autoantibodies in CE using murine and human antigens [33]. Conversely, a retrospective study additionally evaluating paired CE CSF and serum samples detected CSF-specific immunoglobulin G-type oligoclonal bands in 21% of CE cases with drug resistance. However, no evidence of association with ASM response could be detected [106]. These outcomes may reflect the true limited importance of autoimmune encephalitis in CE and/or methodological limitations due to non-homologous antigen use.

#### 4.4.2. Human Epilepsy

In humans, IL-17A promotes hippocampal damage, disrupts BBB integrity, and contributes to epileptogenesis [107]. Certain ASM (carbamazepine, levetiracetam) can also influence coagulation parameters, including D-dimer, PT, and APTT [108].

Regarding autoantibodies, PWE shows variable prevalence among patients. Well-characterized antibodies are primarily observed in human autoimmune encephalitis (e.g., anti-NMDAR, LGI1, CASPR2) [109]. Notably, in cats, an LGI1-autoantibody limbic encephalitis, paralleling its human counterpart, is recognized [110], while the role for autoimmune encephalitis in CE is limited. Human non-encephalitic epilepsy cohorts on the other hand often exhibit low prevalence of antibodies, influenced by antigen selection and laboratory techniques [111].

#### 4.4.3. General Remarks on Immune System Involvement

In recent years, research has strengthened the hypothesis that immuno-inflammatory mechanisms, including both the innate and adaptive immune system, contribute to both the onset of epileptic seizures as well as epileptogenesis [112]. In this context, the complement cascade, pro-inflammatory cytokines and T-cells, and neuronal autoantibodies have been investigated. Observations in CE and PWE indeed highlight a role for the immune system in epilepsy. However, specific pathways, like the complement cascade [24], appear to diverge between species, whereas IL-17 may be relevant in both, although in CE, only one level III study has been described, with variability in ASM management [34].

## 5. Microbiota–Gut–Brain Axis

The microbiota–gut–brain axis (MGBA) provides a bidirectional communication pathway between the gastrointestinal (GI) tract, the enteric nervous system (ENS) and the central nervous system (CNS) via the vagal and spinal afferent nerves, the GI immune system, the hypothalamic–pituitary–adrenal cortical axis and bacterial metabolites in the circulation [113]. The GI microbiota, i.e., the collection of microorganisms residing in the GI tract, represent an important factor within this axis [114]. The MGBA is moreover greatly influenced by nutrition in both dogs and people [19,115]. Recently, interest in the MGBA in the context of epilepsy has been increasing rapidly, both in human [116] and veterinary medicine [117,118]. The pathways involved in the MGBA are not yet fully elucidated, but important interactions with oxidative stress, inflammatory and immune pathways are recognized.

### 5.1. Canine Epilepsy

Studies in CE related to the MGBA have primarily studied and identified microbial changes. Overlapping findings have been detected, although all studies relied on 16S rRNA sequencing, which is inherently prone to interstudy variability caused by sample preparation as well as the bioinformatics and taxonomic databases used [119,120,121]. Hereby, CE showed lower fecal *Prevotella* spp. and *Phascolarctobacterium* [41,42,44,122], while an additional increase in fecal *Escherichia-Shigella* and *Cl. sensu stricto 1* was noted compared to healthy dogs [41,44]. Importantly, the functional impact of the GI microbiota on host metabolism is expected to be more relevant than compositional changes alone. In this context, metabolomics provides a powerful approach to elucidate these functional interactions [123]. This has been implemented in CE too, revealing alterations in histamine and tryptophan metabolism likely related to changes in the GI microbiota, in addition to the tryptophan and kynurenine metabolites discussed earlier (Figure 2). In one CE population studied, fecal indole-3-carboxylic acid, which is a bacterial metabolite of tryptophan, was reduced [41], together with reduced plasma 2,6-dihydroxybenzoic acid [38], which is a diet-associated metabolite microbially derived from phenolic compounds [124]. These findings suggest a different intestinal microbial function in CE.

### 5.2. Human Epilepsy

Previously, the structural and functional similarity of the canine and human GI microbiota, as well as similar reactions to diet, were shown [19]. Indeed, PWE showed some overlapping GI microbiota alterations with CE. Two studies identified decreased fecal *Phascolarctobacterium* in PWE compared to healthy controls [125,126], while an increase in fecal *Escherichia-Shigella* was additionally noticed [125], paralleling the findings in CE. In contrast, another study identified lower fecal *Escherichia-Shigella* and higher *Prevotella* spp. in PWE compared to healthy controls [127], while the same research group later identified a restorative effect of ketogenic diet in children with epilepsy on the GI microbiota composition, including increased fecal *Alloprevotella* spp. [128]. The *Alloprevotella* genus is closely related to *Prevotella* and exerts similar functions, like the production of short-chain fatty acids [129]. Additionally, an earlier diet intervention trial in children with epilepsy revealed increased fecal *Prevotella* spp. following a ketogenic diet [130]. Like for many bacterial genera, considerable functional species and even subspecies strain-level variation exists within the genus *Prevotella* [129], which could explain these apparent contrasting findings in different epilepsy studies.

Additional alterations, not paralleled (yet) in CE, have been detected in PWE. A Mendelian randomization study detected a causal relationship with increased epilepsy risk for bacteria from the Class *Betaproteobacteria* and Order *Burkholderiales* [131], while the elevation of fecal bacterial genera promoting neuroinflammation in PWE was additionally highlighted in a recent meta-analysis [132]. These bacterial alterations highlight the relevance of the MGBA in the pathophysiology of PWE, which is, at least partially, mirrored in CE.

### 5.3. Pre-Clinical Rodent Models

Different rodent studies further established a role for the GI microbiota in epileptogenesis and seizure susceptibility. One study in mice receiving a ketogenic diet, i.e., a low-carbohydrate and high-fat diet resulting in the production of ketone bodies [133], showed that the anticonvulsive effect of this diet was mediated by GI microbiota alterations in *Akkermansia* and *Parabacteroides* [134]. Similarly, another preclinical study showed that fecal microbial transplantation (FMT) from stressed to non-stressed rats accelerated kindling and increased the duration of induced seizures, while, vice versa, fecal transplants from non-stressed to stressed rats could counteract the proepileptic effects of stress [135]. Lastly, in a rat model for posttraumatic epilepsy, the pre-existent fecal microbial abundancies of specific members of the *Lachnospiraceae* family could predict the risk for developing epilepsy following traumatic brain injury [136].

### 5.4. General Remarks on Microbiota–Gut–Brain Axis

Studies show a potentially important role for GI microbiota in the pathogenesis and management of epilepsy. Differences in GI microbiota composition have been demonstrated in CE [41,42,44] and PWE [132] compared to healthy controls. Moreover, interventions altering the composition of the GI microbiota, aiming to reduce the epileptic seizure frequency, have been described in CE [117] and PWE [132]. These findings support a potential role for dysbiosis, i.e., disease-promoting imbalance in the GI microbiota composition [137], in epileptogenesis despite the fact that inconsistent GI microbiota alterations were revealed in CE and PWE. These inconsistencies between studies likely reflect technical limitations of 16S rRNA sequencing data, in addition to potential biological variation. Moreover, the GI microbiota of drug-resistant PWE was significantly altered compared to drug-sensitive PWE, indicating a potential role for the normalization of the GI microbiota towards that of healthy individuals in the management of epilepsy [128,138].

A recent metabolomics study in pediatric epilepsy additionally highlighted a role for tryptophan metabolism and the MGBA by identifying lower plasma indole levels, another microbial tryptophan metabolite, in children with epilepsy compared to age-matched healthy controls [5]. Notably, in CE, higher fecal indole was associated with reduced seizure frequency, potentially revealing similar mechanisms [41]. In children with cerebral palsy, on the other hand, increased fecal indole concentrations were observed in drug-resistant cases [139]. Together, these findings seem to support a role for tryptophan metabolism as a link between the GI microbiota and the brain [140] in both CE and PWE, with potential species- and epilepsy subtype-specific metabolite alterations.

### 5.5. Peripheral Histamine

While peripheral histamine involvement could be hypothesized for CE, based on the increased fecal histamine in these dogs [41], human studies on this remain limited. The existing literature for PWE primarily focuses on central histaminergic signalling, including one study showing higher 1-methylhistamine in the brain tissue of humans with temporal lobe epilepsy [141] and case reports or small-scale clinical studies investigating the effects of histamine receptor antagonists [142]. Two rat models indicate a seizure protective effect for histamine and its precursor [143,144]. Generally, a complex interaction between central histamine, neural excitability and epilepsy exists, whereby the effect is mediated by the location and type of receptors (H_1,2,3_ or H_4_-R) [142]. However, histamine levels in feces are unlikely to be related to the central histaminergic system. Interestingly, high GI concentrations of histamine have been shown to disrupt tight junctions, and thus hamper intestinal barrier integrity [145,146]. Therefore, the findings in CE are suspected to be related to an indirect effect via inflammatory signalling and the MGBA, while this has not (yet) been detected in PWE.

## 6. Oxidative Stress

Oxidative stress is defined as an imbalance between oxidants and antioxidants in favour of the former, leading to disruption of redox signalling and control and/or molecular damage [147]. It is closely associated with mitochondrial function and plays a significant role in the pathogenesis of CNS disorders such as Parkinson’s disease, Alzheimer’s disease, Huntington’s disease, Friedreich’s ataxia, and amyotrophic lateral sclerosis [147]. Moreover, oxidative stress has been implicated in the mechanisms underlying epilepsy and epileptogenesis in a rat model [148].

### 6.1. Canine Epilepsy

In veterinary neurology, recent studies have documented an altered serum oxidative profile in CE compared to healthy controls, with increased levels of advanced oxidation protein products (AOPP) and reduced antioxidant markers such as thiol groups (R-SH), glutathione, paraoxonase-1 (PON-1), and butyrylcholinesterase (BChE) activity [47]. A more recent CE metabolomic study confirmed significant alterations in oxidative stress–related plasma metabolites, i.e., elevated gluconic acid and xanthurenic acid, and decreased carnosine and 2,6-dihydroxybenzoic acid [38]. Consistently, Yonezawa et al. (2024) [45] reported significantly increased concentrations of nitric oxide metabolites (nitrite + nitrate; NOx) in CSF for CE and meningoencephalitis of unknown origin (MUO) compared to healthy controls. In contrast, plasma NOx levels did not differ significantly among healthy controls, CE, and MUO [45].

### 6.2. Human Epilepsy

In PWE, increased AOPP levels, as seen in CE, were associated with increased ROS and myeloperoxidase activity [149]. Notably, AOPP levels have been shown to decrease following the surgical resection of epileptogenic foci in humans [150] further supporting their role as potential biomarkers. The role for lipid peroxidation in PWE, like CE, shows inconsistent results [151,152,153], suggesting the need for more sensitive or specific lipid peroxidation markers in epilepsy research. In addition, natural antioxidants like resveratrol, N-acetylcysteine, and sulforaphane have shown neuroprotective and anticonvulsant properties in PWE and rodent models [153,154,155].

### 6.3. General Remarks on Oxidative Stress

Oxidative stress, with AOPP emerging as potential consistent biomarker between species, may represent an area of convergence in the pathophysiology of CE (one level III study) and PWE. A primary limitation of current evidence is the lack of established causation, as most studies, including all CE studies, are observational and cannot definitively determine if oxidative stress is a driver or a consequence of epileptogenesis.

### 6.4. Oxidative Stress and Antiseizure Medication

Although oxidative stress is not considered a primary mechanism of action for currently available ASM, several studies demonstrate that ASM can modulate redox pathways. Valproate, for example, has been shown to reverse glutathione depletion and lipid peroxidation in PTZ-induced rodent seizure models [156,157,158,159]. Similar antioxidant activity has been observed for phenytoin, phenobarbital, carbamazepine [159], lamotrigine [160] and diazepam [161]. Therefore, studies on oxidative stress in CE and PWE should be interpreted with caution, given the often-variable ASM management in the studied populations.

## 7. Lipid Metabolism

Possible disruptions in lipid metabolism in epilepsy primarily involve triglycerides, cholesterol, sphingolipids, and fatty acids [162]. In CE, one study identified significantly higher fasting serum triglyceride concentrations in ASM-treated dogs [46]. Likewise, several observational studies in PWE have documented elevated plasma triglyceride levels in subsets of patients, particularly those with drug-resistant epilepsy, suggesting a potential association between lipid dysregulation and drug resistance [163,164]. However, such associations may be confounded by the metabolic effects of chronic ASM therapy [165]. In CE, phenobarbital administration has been shown to significantly decrease the fecal abundance of *Clostridiales* and increase fecal SCFAs [166].

Growing evidence supports a role for SCFAs, including acetate, propionate, and butyrate, in mechanisms relevant to epilepsy. Supporting this hypothesis, fecal supernatants from dogs with idiopathic epilepsy have been demonstrated to activate enteric neurons, suggesting that microbiota-derived metabolites, potentially including SCFAs, may exert direct neuroactive effects along the MGBA [167]. Experimental studies in rodent models indicate that reduced SCFA availability is associated with increased seizure susceptibility, whereas supplementation with specific SCFAs, particularly butyrate, can exert anticonvulsant and neuroprotective effects [135]. Consistent with these findings, both PWE and CE exhibit alterations in gut microbiota composition that may affect SCFA-producing taxa, as discussed in the preceding sections [41,128,138,168].

Both in PWE and CE, dietary interventions that modify triglyceride composition, particularly ketogenic diets or those based on medium-chain triglycerides, have shown potential antiepileptic benefits and an acceptable safety profile [18].

### General Remarks on Lipid Metabolism

Lipid metabolism dysregulation is increasingly recognized as a relevant component of epilepsy pathophysiology in both PWE and CE, involving multiple lipid classes and interacting with oxidative stress, inflammatory pathways, and gut–brain axis signalling [46,162,163,164]. Although alterations in triglycerides, cholesterol, sphingolipids, and fatty acids have been reported across studies, their causal contribution to epileptogenesis remains uncertain and may be influenced by antiseizure medication effects as well as other metabolic confounders [46,163,165,166,167]. In addition, emerging evidence linking short-chain fatty acids to neuronal excitability suggests a potential mechanistic link between microbiota-derived metabolites and seizure susceptibility, although current data remain largely indirect [36,135,164,169]. Dietary lipid-modifying interventions, including ketogenic and medium-chain triglyceride diets, further support a functional role of lipid metabolism in seizure modulation in both species [18].

Overall, CE mirrors several lipid-related alterations observed in PWE and well-controlled mechanistic studies are required to clarify causal pathways and the therapeutic relevance of lipid metabolism in epilepsy. In this context, the dog represents a valuable translational model for investigating lipid-mediated neuronal mechanisms and for refining therapeutic strategies [18], owing to the feasibility of dietary standardization combined with environmental exposure patterns comparable to those of PWE.

These findings support the presence of overlapping alterations in triglyceride metabolism and SCFA-associated pathways in CE and PWE, while some discrepancies across studies may indicate species-specific metabolic responses and differential effects of ASM treatment and diet.

## 8. Amino Acid and Protein Metabolism

### 8.1. Protein Metabolism

#### 8.1.1. Amyloid-β

In drug-resistant CE, systemic alterations in amyloid metabolism have been reported, as evidenced by significantly increased plasma β-amyloid (Aβ) 42 concentrations compared with healthy dogs [36]. Similarly, elevated levels of Aβ have been associated with epilepsy in PWE, as well as in murine models [36,169,170]. Furthermore, an early-onset canine neurodegenerative disorder associated with PITRM1 (a mitochondrial protease involved in peptide degradation) dysfunction has been described, characterized by mitochondrial impairment, Aβ accumulation, and fatal epilepsy, although this is currently only reported in a single breed with a specific epilepsy syndrome [171]. Moreover, it has been hypothesized that the Aβ precursor protein contributes to the pathophysiological mechanisms underlying drug-resistance in PWE, supported by its increased expression in temporal lobe and hippocampal tissue from drug-resistant PWE [170,172]. Recent evidence suggests that elevated Aβ levels in adulthood represent a risk factor for late-onset epilepsy, while amyloid pathology also correlates with cognitive impairment in PWE [169]. In line with these clinical observations, in APP/PS1 transgenic mice, the presence of Aβ plaques has been correlated with an increased frequency and duration of epileptiform discharges, further reinforcing the link between Aβ pathology and seizure susceptibility [173].

The increase in Aβ levels in both species may explain the observed correlation of PWE and CE with Alzheimer’s disease in humans and its equivalent in dogs: canine cognitive dysfunction (CCD) syndrome [174,175]. In humans, epileptic seizure activity itself has been shown to trigger neurodegenerative processes by generating aberrant electrical currents and promoting both the production and release of Aβ [173,176]. Moreover, the presence of Aβ42 has been demonstrated to enhance neuronal excitability in a rodent model of Alzheimer’s disease, thereby facilitating the development and progression of epilepsy [177]. Canine studies have further supported these associations. The dynamics of plasma Aβ concentrations in dogs mirror those observed in ageing humans, both with and without AD [178,179]. Additionally, dogs affected by drug-resistant epilepsy have been shown to exhibit an increased risk of developing CCD at a younger age compared with neurologically normal dogs [180].

#### 8.1.2. Haptoglobin

Baka et al. conducted two distinct studies on CE, performing proteomic analyses on both serum and CSF from the same cohort. Epileptic dogs were divided into three groups: CE treated with ASM, untreated CE, and structural epilepsy. Comparison with healthy dogs revealed significant alterations in both CSF and serum across all groups for several proteins, including haptoglobin (HP) [35,37]. The multifunctional protein HP exhibits anti-inflammatory properties that facilitate Aβ clearance [181,182] and exerts antioxidant effects by binding free hemoglobin, thereby preventing oxidative tissue damage [183]. Its role in epilepsy remains to be fully elucidated, as current evidence in PWE remains inconsistent. Previous studies in PWE have reported an association between reduced plasma HP levels and the occurrence of epileptic seizures [184,185,186], whereas others have shown increased HP levels associated with refractory epilepsy in children [187] and idiopathic epilepsy in adults [186]. Conversely, some investigations have found no significant association between HP and seizure occurrence [188]. Experimental evidence suggests that decreased HP may impair the clearance of free hemoglobin in the CNS, potentially contributing to epileptogenesis, while elevated HP may reflect immune activation and BBB dysfunction [186,189].

#### 8.1.3. Matrix Metallopeptidase 2

The zinc-dependent endopeptidase MMP-2, which is decreased in treated CE, is known to be involved in extracellular matrix degradation and BBB integrity [190,191]. It also exhibits anti-inflammatory effects via interactions with interleukin-4 and interleukin-13 [192] and is upregulated in various neurological and inflammatory disorders in humans [190,193,194], as well as in human non-infectious conditions with an inflammatory component [195,196]. However, MMP-2’s role in epileptogenesis remains unclear [197]. In the studies by Baka et al. mentioned above, downregulation of MMP-2 was observed in the CSF of treated dogs with CE. This reduction may reflect the effects of ASM, the time elapsed since the last epileptic seizure, or excessive utilization with slow resynthesis [35].

### 8.2. Amino Acid Metabolism

Parallel to the proteomic studies, canine metabolomics research revealed alterations in amino acids comparing CE and healthy dogs in multiple biofluids, i.e., CSF, plasma and feces [38,39,41,198]. Notably, disruptions in the levels of lysine, threonine, leucine, methylated and acetylated amino acids, phenylalanine and glutamate were identified in plasma and CSF using a targeted approach [38,39]. Importantly, CSF metabolic differences between idiopathic and structural epilepsy in dogs highlight an impact of the pathophysiological mechanism beyond the occurrence of epileptic seizures [39]. Additionally, altered urinary levels of glycine and serotonin, as well as a decreased γ-aminobutyric acid (GABA)–glutamate ratio, have been reported in CE [51]. Similar metabolic disturbances have been identified in PWE and other animal models, including disruptions in alanine, aspartate, and glutamate metabolism, together with disruptions in glycine, serine, and threonine metabolism [199,200,201].

Analyzing individual amino acids in greater detail across studies reveals both convergent and divergent metabolic alterations associated with epilepsy. A human Mendelian randomized study suggested a causal impact on PWE for increased leucine [202]. This might align with the higher plasma leucine detected in CE [38]. However, from a therapeutic perspective, in PWE, leucine, together with lysine, could enhance a ketogenic diet’s ability to treat epileptic seizures [203]. For lysine, an imbalance in acetylation and deacetylation has been described in PWE [204]. Additionally, lysine metabolism disorders, such as SEDT1B-related syndrome, are associated with epilepsy in humans [205]. In CE, an increase in plasma N_6_-acetyl-lysine [38] might be linked with a similar pathophysiological mechanism. Hypothesized mechanisms causing dysregulation of protein lysine acetylation include altered deacetylase activity or increased mitochondrial acetyl-CoA [206,207].

In contrast, threonine alterations appear to differ between species and disease states. While threonine levels were reported to be increased in CE, serum threonine concentrations were decreased in PWE compared with healthy controls [208]. Moreover, a transient reduction in serum threonine has been observed following seizures in PWE, whereas no significant changes were detected under basal conditions [209]. These metabolic discrepancies may be species-specific or related to the time elapsed since the last epileptic seizure. The latter could be supported by observations in a rodent model of temporal lobe epilepsy, which demonstrated distinct alterations in glycine, serine, and threonine metabolism at 48 h (i.e., increased glycine) versus 6 weeks (i.e., no significant changes in glycine) after status epilepticus [210].

#### Tryptophan

As addressed in the preceding sections and schematically illustrated in Figure 2, tryptophan metabolism (particularly the kynurenine pathway) lies at the interface of inflammation, oxidative stress, and the microbiota–gut–brain axis. Comparative evidence from canine and human studies consistently highlights the involvement of this pathway in epilepsy across species.

The kynurenine pathway generates bioactive metabolites that modulate immune responses and neuroactive signalling, thereby linking peripheral metabolic alterations to CNS function. Alterations in tryptophan–kynurenine metabolism have been reported in adults with status epilepticus [211], PWE [208], drug-resistant epilepsy [212], and children with epilepsy [5]. A metabolic shift toward increased kynurenic acid production has been described in children with drug-resistant epilepsy responding to a ketogenic diet [213], while elevated fecal kynurenic acid levels were also reported in children with cerebral palsy and epilepsy [139].

Beyond kynurenine pathway metabolites, serotonin, a major downstream product of tryptophan metabolism, has also been implicated in epilepsy. Serotonin exists in two functionally and anatomically distinct pools: a central serotonergic system, synthesized in the brain by tryptophan hydroxylase 2 (TPH2), and a peripheral serotonergic system, accounting for ~90–95% of total body serotonin and predominantly produced in gastrointestinal enterochromaffin cells via tryptophan hydroxylase 1 (TPH1) [214,215]. Peripheral serotonin does not cross the blood–brain barrier; therefore, changes in fecal, urinary, or circulating serotonin primarily reflect gut-derived and microbiota-modulated tryptophan metabolism rather than direct alterations in central serotonergic neurotransmission [215].

While central serotonergic mechanisms are discussed in detail in the Neurotransmission section, accumulating evidence supports a relevant role for peripheral serotonin dysregulation in epilepsy. In dogs, fecal serotonin was increased in CE with a mild epileptic phenotype, whereas fecal indole-3-carboxylic acid was decreased in both mild and drug-resistant dogs compared to healthy dogs [41]. Additionally, altered urinary serotonin levels further support the dysregulation of downstream tryptophan metabolism in CE [51].

In humans, changes in platelet serotonin content, serum serotonin levels, and urinary serotonin metabolites have been reported in PWE and have been linked to seizure burden, autonomic dysfunction, and systemic inflammatory states [216,217]. In addition, peripheral serotonergic dysregulation has been implicated in the pathophysiology of sudden unexpected death in epilepsy, highlighting the relevance of non-central serotonin pools in epilepsy-related morbidity [216]. Collectively, the current data support a potential role of peripheral serotonin dysregulation as part of the broader alterations in tryptophan metabolism observed in PWE and dogs with CE.

Finally, several vitamins and minerals involved as enzymatic cofactors in tryptophan metabolism, including vitamin B6, pantothenic acid, magnesium, iron, and zinc, have been reported to be altered in both CE and PWE, potentially contributing to the dysregulation of downstream metabolic pathways [218].

### 8.3. General Remarks on Amino Acid and Protein Metabolism

Collectively, comparative evidence from CE and PWE indicates convergent alterations in amyloid metabolism, amino acid homeostasis, and tryptophan–kynurenine pathway signalling, supporting shared mechanisms linking neuroinflammation, neuronal excitability, and gut–brain axis dysfunction across species. Nevertheless, differences in specific metabolites, including threonine and other amino acid profiles, may reflect species-specific metabolic responses as well as variability related to seizure timing and ASM exposure.

## 9. Minerals, Trace Elements and Vitamins

### 9.1. Canine Epilepsy

Evidence for the involvement of vitamins in CE is limited; however, one study mentioned vitamin B6 as a key metabolite, with markedly reduced plasma concentrations observed in CE compared to healthy dogs [38]. No other studies evaluating vitamins in CE could be retrieved. Conversely, minerals and trace elements have been evaluated in different CE studies. First, a study by Vitale et al. (2019) reported significantly elevated serum copper, manganese, selenium, and zinc concentrations in CE [50] compared to healthy controls. Increased micromineral levels were subsequently corroborated by Rosendahl et al. (2023), who demonstrated higher whole-blood concentrations of copper and selenium, an increased Cu/Zn ratio, and lower whole-blood chromium concentrations in CE [48]. Additional evidence from one study assessing trace element concentrations in the hair of dogs with CE further supports the occurrence of such alterations [49]. From these studies, blood copper and hair arsenic concentrations seem to be linked to ASM rather than epilepsy. Notably, only selenium concentrations in CE were above the established reference range for healthy dogs in all sample types. Surprisingly, negative long-term health effects have solely been associated with selenium deficiency rather than toxicity [219,220]. Therefore, future studies should include markers for selenium bioactivity and urinary excretion to provide insights into the biological meaning of selenium alterations in CE.

### 9.2. Human Epilepsy

#### 9.2.1. Copper and Zinc

In PWE, elevated blood copper levels have been reported, with some evidence linking this increase to specific ASM [221,222,223]. Phenobarbital therapy, for instance, can enhance ceruloplasmin oxidation, thereby elevating serum ceruloplasmin and copper concentrations [224]. Importantly, the Cu/Zn ratio has been considered a more sensitive biomarker than the individual mineral concentrations, with significant increases documented in children with epilepsy compared to healthy controls [221,225]. However, it should be noted that these measurements are limited to the blood, which may not always accurately reflect tissue mineral status due to the body’s homeostatic regulation [226].

Copper plays an essential role in brain health, contributing to neurotransmitter synthesis, synaptic activity modulation, and nerve myelination. Both Cu deficiency and excess can have deleterious effects on neuronal integrity and function [227]. Excess Cu can enhance reactive oxygen species (ROS) production, despite the antioxidant properties of Cu-Zn superoxide dismutase, triggering pro-inflammatory responses and potentially increasing the risk of neurotoxicity and epilepsy [228]. Conversely, Cu deficiency can impair mitochondrial respiration, reduce antioxidant defence, and disrupt neurotransmitter metabolism, ultimately leading to neuronal dysfunction and enhanced seizure susceptibility [229,230].

Zinc, on the other hand, exhibits antioxidant and anti-inflammatory properties, and the Cu/Zn ratio has been proposed as a biomarker of oxidative stress and inflammation [231,232], both of which, as discussed previously, are implicated in epileptogenesis. Interestingly, zinc supplementation has been associated with reduced epileptic seizure frequency in PWE, while seizure activity returned upon withdrawal of supplementation [233].

#### 9.2.2. Selenium

Selenium status in PWE remains a subject of debate. One recent meta-analysis reported reduced selenium concentrations in PWE [234,235], whereas another study conversely found significantly higher levels in PWE compared with controls [235]. Earlier studies in pediatric patients with intractable epilepsy additionally showed low serum selenium concentrations [233], and moreover, clinical improvements have been reported in selenium-deficient PWE following supplementation [236,237,238]. Such discrepancies may be related to selenium’s narrow safety margin in humans, unlike dogs [239,240]. Indeed, impaired selenoprotein expression, reduced selenium availability, disruption of sodium selenate biosynthesis, or impaired brain selenium transport have been implicated in the pathogenesis of epilepsy and other neurodevelopmental disorders [241]. Conversely, excessive selenium can promote oxidative stress through glutathione depletion, suppress cholinergic signalling, and induce cholinergic neuron degeneration [242]. Maintaining optimal selenium levels is critical to harness its antioxidant benefits without incurring neurotoxic risks [243].

#### 9.2.3. Vitamin B6

**Figure 3:**
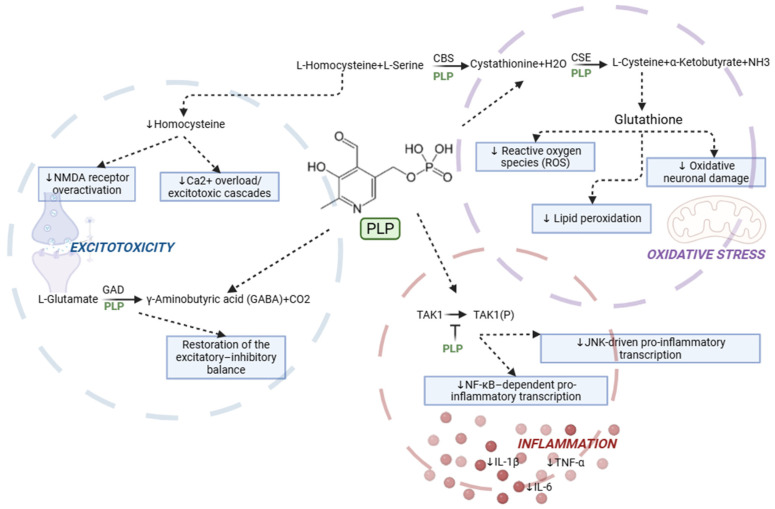
Schematic overview of pyridoxal-5′-phosphate (PLP)-dependent pathways suggested in modulating the neurotoxic triad underlying epileptogenesis. Solid arrows indicate biochemical reactions or metabolic conversions, with PLP labels highlighting PLP-dependent enzymatic steps; dashed arrows indicate proposed indirect mechanistic links or downstream effects; and blunt-ended lines indicate inhibitory modulation. Downward arrows indicate a reduction in the indicated process or mediator. PLP: pyridoxal-5′-phosphate; NMDA: N-methyl-D-aspartate; GAD: glutamate decarboxylase; CBS: cystathionine β-synthase; CSE: cystathionine γ-lyase; TAK1: transforming growth factor-β-activated kinase 1; TNF: tumour necrosis factor; NF-κB: nuclear factor kappa B; JNK: c-Jun N-terminal kinase; IL: interleukin. Created in BioRender.com.

The current literature suggests that vitamin B6, more specifically its active form pyridoxal-5′-phosphate (PLP), may modulate the neurotoxic triad of excitotoxicity, oxidative stress, and inflammation that underlies epileptogenesis [244], as schematically illustrated in Figure 3. First, PLP is hypothesized to restore the excitatory–inhibitory balance by facilitating the conversion of glutamate into GABA [245,246,247]. Second, PLP may reduce homocysteine levels, theoretically preventing NMDA receptor overactivation and excessive neuronal excitation [248,249]. Third, PLP is proposed to enhance glutathione synthesis, thereby protecting neurons from oxidative damage [250,251,252,253]. Lastly, PLP has been suggested to enhance epileptic seizure control by modulating cytokine production, exerting anti-inflammatory effects [254,255].

However, a water-soluble, high-dose administration of vitamin B6 can additionally cause reversible neurological side effects in humans and dogs [256,257]. Therefore, optimal dosing would be primordial to attain therapeutic benefits. The levels of PLP may be influenced by ASM in PWE, although findings vary across drug types and study designs [258,259,260,261,262]. Most studies report significantly lower PLP levels in PWE, particularly in those receiving enzyme-inducing ASM, like phenytoin or carbamazepine [261,262].

### 9.3. General Remarks on Vitamins, Minerals and Trace Elements

Emerging research highlights that trace elements, particularly selenium, iron, copper and zinc, are associated with both the development of epilepsy and changes in seizure susceptibility in PWE, CE and rodent models [263]. Antiseizure interventions, including ASM and the ketogenic diet, have been shown to modify the serum concentrations of these elements, suggesting that the maintenance of trace element homeostasis may play a role in both the prevention and the therapeutic management of epilepsy [264]. In addition to trace elements, several vitamins have also been implicated in PWE, with deficiencies or imbalances potentially influencing neuronal excitability and seizure thresholds [265,266]. In CE, vitamin B6 remains the only vitamin directly linked to epilepsy in one level II study, while the status and potential involvement of other vitamins have not yet been systematically investigated in this population [38].

## 10. Neurotransmission

### 10.1. Canine Epilepsy

Urinary neurotransmitter profiling in CE revealed a decreased GABA/glutamate and norepinephrine/epinephrine (NE/E) ratio [51]. The same study further indicated that sex and ASM therapy may modulate these concentrations [51]. Furthermore, two earlier studies investigated neurotransmitter alterations in CE. Among these, Ellenberger et al. (2004) confirmed altered CSF concentrations of GABA and glutamate, as well as changes in their ratio [52]. Similarly, Morita et al. (2005), employing cerebral microdialysis, electroencephalographic recordings, and immunohistochemical analyses, documented elevated extracellular concentrations of glutamate and aspartate in CE brains, which were associated with an increased spike frequency [53].

### 10.2. Human Epilepsy

Although the function for GABA and glutamate in maintaining the brain’s inhibitory–excitatory balance is well established, studies in PWE on glutamate and GABA are limited to small cohorts with suboptimal statistical power, and an incomplete understanding of how central and peripheral concentrations relate to each other [201]. The observation of analogous neurochemical alterations in CE suggests that dogs may constitute a translational model for investigating glutamate–GABA dysregulation and guiding the development of targeted interventions [38,201].

### 10.3. Glutamate and Glutamic Acid

Across human and animal studies, altered glutamate and glutamic acid levels have been consistently documented in the blood [38,200,209] and CSF [39,201,267,268,269] of individuals with epilepsy. Under physiological conditions, excess glutamate is actively cleared from the CNS into systemic circulation. However, the relationship between central and peripheral glutamate is complex: while blood glutamate concentrations may partially reflect brain metabolism, they are also influenced by peripheral sources (e.g., muscle, liver, and gut) and by the selective transport properties of the blood–brain barrier [270,271]. As the primary excitatory neurotransmitter in the CNS [271], glutamate accumulation is central to epileptogenesis. Perturbations in the excitatory–inhibitory equilibrium, particularly altered GABA–glutamate ratios, constitute a core mechanism underlying seizure initiation [201]. Beyond its central actions, glutamate also serves as an excitatory neurotransmitter within the enteric nervous system, where enteric neurons express glutamatergic receptors and transporters analogous to those found in the central nervous system [272,273]. Glutamatergic signalling along the MGBA has been proposed as a mechanism by which metabolic and neuroactive signals in the periphery may influence brain function [274]. Furthermore, studies in rodent models indicate that gastric glutamate can activate afferent pathways that are suppressed by vagotomy, consistent with the vagal modulation of central responses to gut stimuli [275].

### 10.4. Epinephrine and Norepinephrine

Epinephrine and norepinephrine are closely linked to epilepsy in both humans and dogs. In PWE, noradrenergic system dysregulation has been hypothesized to be correlated with common comorbidities such as sleep disturbances and cognitive deficits [276,277,278,279]. Similarly, in CE, increased urinary epinephrine concentrations likely cause the altered urinary norepinephrine/epinephrine ratio [51] and, like in PWE, these are associated with common comorbidities, including sleep disturbances, ADHD-like behaviours, and anxiety [51,180,280,281].

### 10.5. Glycine

Glycine is an inhibitory neurotransmitter that, when dysregulated, can induce epileptic seizures through multiple mechanisms at both low and high concentrations [282,283]. Elevated urinary glycine levels detected in CE may be associated with seizure onset, cognitive impairment, and hyperactivity, consistent with clinical observations in PWE [51,180,284,285]. Evidence from human and murine studies indeed indicates that dysfunction in glycine receptors is associated with epileptic phenotypes, and several receptor subtypes have been proposed as potential therapeutic targets [286,287,288,289]. Further evidence supporting the involvement of glycine in epilepsy pathogenesis comes from the existence of a hereditary disorder of glycine metabolism in humans, known as nonketotic hyperglycinaemia [290]. This condition leads to the accumulation of glycine in the body, resulting in refractory epileptic seizures, hyperactivity, elevated levels of glycine in the urine, and, in adults, cognitive impairment [291]. In contrast to PWE, in whom valproate, an ASM, has been shown to markedly increase both urinary and plasma glycine concentrations [292], no comparable effect has been documented in CE treated with first- or second-line ASM (i.e., phenobarbital or potassium bromide) approved for the management of CE [51].

### 10.6. Serotonin

As mentioned in previous sections, serotonin represents a key downstream product of tryptophan metabolism; however, beyond its peripheral metabolic role, it also exerts critical functions as a central neurotransmitter relevant to epilepsy. In humans, reduced serotonin levels are associated with various psychiatric disorders. Furthermore, in PWE, alterations in the serotonergic system have been shown to lower the epileptic seizure threshold and contribute to frequently co-occurring neurobehavioral comorbidities [293,294]. The International League Against Epilepsy has suggested that selective serotonin reuptake inhibitors (SSRIs) may be used with caution for the treatment of anxiety in certain PWE. These drugs are already widely used to manage behavioural and psychological disorders in both species [284,295,296].

Experimental evidence in PWE indicates that SSRIs or serotonin–norepinephrine reuptake inhibitors (SNRIs) can reduce the severity of epilepsy by alleviating depressive symptoms [297,298]. Additionally, in dogs, fluoxetine (an SSRI) has been reported to be effective in treating fly-snapping syndrome, a condition considered to represent limbic epilepsy by some but a compulsive behavioural disorder by others [299].

### 10.7. General Remarks on Neurotransmission

Overall, converging evidence in both PWE and CE supports a consistent dysregulation of excitatory and inhibitory neurotransmission, particularly involving glutamatergic, GABAergic, glycinergic, and monoaminergic systems [38,39,200,201,209]. Despite this general agreement, the current findings are limited by small sample sizes, methodological heterogeneity, and an incomplete understanding of the relationship between central and peripheral neurotransmitter levels [201,270,271]. The observed similarities between species may further support the translational relevance of CE as a natural model of epilepsy [38,201]. Nevertheless, causal mechanisms remain largely undefined, and future studies with standardized approaches and appropriate control of confounders, including ASM, are required to better define the role of neurotransmitter alterations in epileptogenesis [38,51,201,282].

## 11. Endocannabinoid System

### 11.1. Endogenous Endocannabinoid Metabolism

The endocannabinoid system is a neuromodulatory network composed of endocannabinoids, like anandamide, 1- and 2-arachidonoyl glycerol, their receptors (CB1, CB2), and associated enzymes, which regulates synaptic transmission and maintains homeostasis in the central and peripheral nervous system. It plays a key role in modulating neuronal excitability, inflammation, and seizure susceptibility [300]. Importantly, species-specific differences in CB1 expression have been documented, particularly regarding regional distribution, cellular localization, and density. In dogs, CB1 is widely expressed in the cerebral cortex, hippocampus, basal ganglia, cerebellum, hindbrain, spinal cord, peripheral nerves, and glial cells [301,302,303], whereas in humans CB1 is predominantly neuronal and concentrated in the hippocampus, cortex, amygdala, basal ganglia, and cerebellum [304,305]. Moreover, CB1R expression in humans is dynamic rather than static: it varies during development, with ageing, and in response to stress or environmental experiences [306]. Therefore, while dogs could serve as a model to study differential receptor modulation between healthy and CE, they are not suitable for the direct comparison of CB1 receptor localization across species.

#### 11.1.1. Canine Epilepsy

In CE, higher CSF anandamide was detected, and this increase was even more pronounced in dogs with a severe phenotype, i.e., status epilepticus and/or cluster seizures, or long disease history [54]. More recently, a distinct modification in the hippocampal CB1 receptors was highlighted for CE compared to structural epilepsy. Dogs with CE showed decreased CB1 receptor expression in the CA1 region, in contrast to dogs with structural epilepsy, showing increased expression [55].

#### 11.1.2. Human Epilepsy

In humans with newly diagnosed temporal lobe epilepsy, decreased CSF anandamide was detected [307]. This seems to contrast with the increased CSF anandamide in CE; however, the authors of the CE study suggest a physiological counter-mechanism to attempt to regulate epileptic seizure thresholds. This would indeed not yet be present in the acute phase of the disease studied in PWE. The alteration in CB1 receptors is considered complex and depending on the etiology in PWE [308], with, e.g., a downregulation in the hippocampus of people with temporal lobe epilepsy [309], similar to the distinct receptor modification seen in CE. However, for CB1 expression no direct cross-species comparison is possible, as stressed by the differences in regional distribution, cellular localization, and density [302].

### 11.2. Phytocannabinoids

Both in PWE and CE, cannabidiol, i.e., a phytocannabinoid, emerged as a novel management strategy, highlighting potential efficacy in epileptic seizure reduction, but also showed adverse effects like somnolence and gastro-intestinal symptoms [310,311]. Most research to date argues against the relevance of CB1 and CB2 receptors in the anticonvulsive effect of phytocannabinoids [312]. Other receptors, including the G protein receptor 55 (GPR55) and transient receptor potential Vannilloid-1 (TRPV1) [313,314], together with an interaction via adenosine signalling [315], are hypothesized as the major pathways leading to its anticonvulsive effects.

### 11.3. General Remarks on the Endocannabinoid System

The endocannabinoid system represents a key neuromodulatory pathway implicated in the regulation of neuronal excitability and seizure susceptibility in both PWE and CE [300]. Evidence from both species indicates alterations in endocannabinoid signalling, including changes in anandamide levels and CB1 receptor expression, although these appear to be context-dependent and influenced by disease type and stage [54,55,307,308]. However, interpretation is limited by interspecies differences in CB1 distribution and regulation, as well as by variability in study design and clinical populations [302,304,305]. While CE shares several biochemical and receptor-level similarities with PWE, direct anatomical and functional comparisons remain constrained. In addition, although cannabidiol shows anticonvulsant effects in both species, current evidence suggests these are largely mediated through non-CB1/CB2 mechanisms [310,311,312,313,314,315]. Further studies are required to clarify the precise role of endocannabinoid signalling in epileptogenesis and its translational therapeutic potential.

## 12. MicroRNA

MicroRNAs (miRNAs) play a crucial role in the post-transcriptional regulation of genes involved in neuronal metabolic pathways, including mitochondrial function, glycolysis, and oxidative stress responses. Emerging evidence indicates that altered miRNA expression contributes to the metabolic dysregulation observed in epilepsy, affecting neuronal energy homeostasis and excitability. These findings suggest that miRNA–metabolism interactions represent a key molecular link between bioenergetic imbalance and epileptogenesis [316].

### 12.1. Canine Epilepsy

A recent study investigated microRNAs (miRNAs) in CE [56]. The study focused on seven miRNAs previously identified as dysregulated in both human and murine epilepsy [317,318,319,320,321]. Six of these (miR-16, miR-27a-3p, miR-93-5p, miR-132, miR-142, and miR-574-3p) were found to be altered in CE, with five showing significant downregulation. Notably, a panel combining miR-93-5p, miR-142, and miR-574 demonstrated promising diagnostic performance [56].

In earlier work on CE, Gutierrez-Quintana et al. (2022) [322] investigated miR-134, a miRNA upregulated in rodent models of drug-resistant epilepsy and in human temporal lobe epilepsy. Plasma levels of miR-134 were significantly higher in drug-resistant CE compared to controls and drug-responsive dogs [322]. Additionally, Pasierbinska et al. (2025) [323] profiled blood miRNAs in drug-naive and treated CE in a pilot study, identifying miRNAs that distinguish CE from healthy dogs (e.g., miR-381, miR-214, miR-224), and those differentiating drug-resistant versus drug-responsive cases (e.g., miR-223, miR-129, miR-210). These results support a potential role of miRNAs in the molecular mechanisms underlying CE and drug-resistance, establishing a basis for further biomarker discovery and therapeutic studies.

### 12.2. Human Epilepsy

In PWE, several miRNAs have been implicated in neuronal plasticity, dendritic spine morphology, synaptic regulation, and excitability. Similar to CE, miR-134 (a neuron-enriched miRNA involved in dendritic spine development through targets such as LIMK1) is consistently upregulated in the brain tissue of patients with drug-resistant temporal lobe epilepsy, reflecting synaptic remodelling associated with hyperexcitability [324,325]. Circulating levels of miR-134 are also elevated in plasma or serum of patients with drug-resistant epilepsy, suggesting potential as a biomarker for pharmacoresistance [318]. Additionally, miR-129-2-3p is upregulated in cortical tissue and plasma of patients with refractory temporal lobe epilepsy. ROC analyses indicate miR-129-2-3p can discriminate PWE from controls with good sensitivity and specificity [325].

For miR-223, findings in PWE are inconsistent across studies, but it has been reported to be overexpressed in the serum of patients with temporal lobe epilepsy and may differentiate drug-resistant from drug-responsive cases. Similarly, miR-210, a hypoxia-regulated miRNA, shows limited and inconsistent evidence in PWE [317,326], with most functional data derived from animal models, where it modulates neuronal survival and GABAergic signalling post-seizure [317].

In summary, studies in PWE strongly support a role for miR-134 and miR-129-2-3p in pathophysiology and drug resistance, while canine evidence remains preliminary. Systematic cross-species studies are needed to establish the translational relevance for biomarker discovery and targeted interventions.

## 13. Limitations

This review is subject to several limitations that warrant critical consideration. Firstly, an inherent selection bias arises from the hybrid review design, wherein the canine literature search was systematic, but the synthesis of the human literature was non-systematic and narrative. This approach introduced a bias towards metabolic pathways and markers previously identified in dogs, rather than providing a comprehensive systematic overview of human metabolic epilepsy research—in line with the proposed research aim. Secondly, the current body of evidence is predominantly associative, with most retrieved studies being observational in nature. Consequently, it remains unclear whether metabolic alterations, such as neuroinflammation or oxidative stress, are primary drivers of epileptogenesis or secondary consequences of recurrent seizures. Thirdly, confounding variables, particularly the influence of ASM management, pose significant challenges. Many studies failed to adequately account for or standardize ASM management, yet ASM may modulate redox pathways, influence lipid profiles, and affect inflammatory markers, leaving their metabolic impact inconclusive. Additionally, variability in dietary intake and fasting conditions at the time of sampling further complicates metabolic research, particularly in human studies, where dietary standardization is difficult. Finally, cohort and methodological limitations, including small and heterogeneous sample sizes might reduce statistical power and generalisability. Technological biases, such as those inherent in 16S rRNA sequencing, introduce interstudy variability, while biological inconsistencies, including the use of non-homologous antigens for autoantibody testing in CE and inconsistent markers for lipid peroxidation, highlight the need for more sensitive and specific analytical tools in epilepsy research for both species.

## 14. Conclusions and Future Insights

The present review underscores a substantial convergence in the metabolic alterations associated with CE and PWE, while acknowledging species-specific divergences that warrant further investigation. Shared inflammatory pathways, including, e.g., increased IL-17 and TNF-α, oxidative stress markers like AOPP, amyloid pathology, and metabolic responses to dietary interventions—especially MCT diets—highlight the translational potential of the CE model. However, differences in complement cascade activation, specific amino acid alterations like threonine, and cannabinoid receptor localisation emphasize the need for the cautious extrapolation of findings between species.

**Figure 4:**
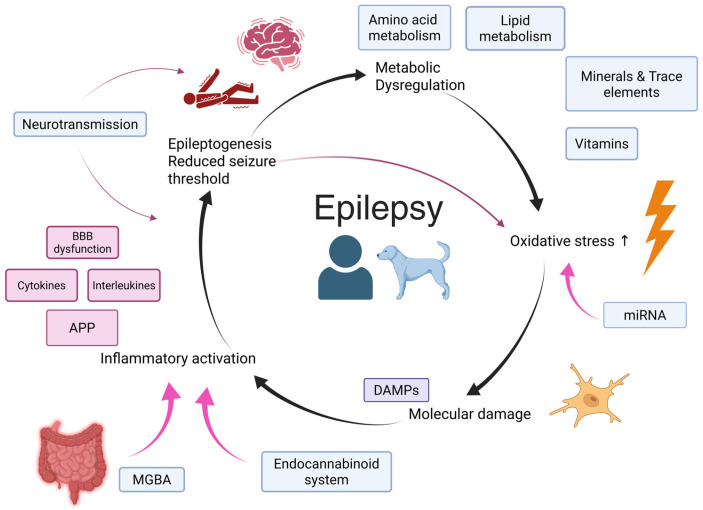
Theoretical self-perpetuating cycle in the pathogenesis of epilepsy in dogs and humans. miRNA: micro-RNA; DAMPs: damage-associated molecular patterns; MGBA: microbiota–gut–brain axis; BBB: blood–brain barrier; APP: acute phase proteins. Created in BioRender.com.

In both CE and PWE, inflammatory and immune pathways, together with mitochondrial function and related oxidative stress, are hypothesized to emerge as central mechanisms in epilepsy and appear to form a self-perpetuating cycle (Figure 4). Metabolic disturbances in amino acids, lipids, minerals, trace elements, and vitamins may act as triggers within this cycle. Additional alterations in the endocannabinoid system, microbiota–gut–brain axis, neurotransmission, and post-transcriptional regulation further appear to contribute to epileptogenesis and may modulate these interactions.

Priorities for future research include: (1) harmonized cross-species, longitudinal cohorts with standardized phenotyping, including epileptic seizure semiology, comorbidities, diet information, and ASM exposure, to address current study heterogeneity and common confounding factors; (2) multi-omics pipelines integrating metabolomics, proteomics, trace-element and vitamin status, microbiome and microRNAs to derive robust biomarker panels and responder stratifiers; (3) randomized, clinical trials of nutritional and metabolic interventions, e.g., the MCT-diet, and GI microbiome modulation (pre- and probiotics, FMT); and (4) the translational validation of emerging targets, like specific miRNAs, immunomodulation, and endocannabinoid regulation, with attention to the species-specific characteristics highlighted in this review. Taken together, CE has the potential to bridge experimental models and clinical practice, enhancing epilepsy research in both species.

## Figures and Tables

**Table 1:** Summary of canine studies identified through scoping literature search.

Reference	Category	Population	Sample Type	Main Findings in CE Dogs	ASM Exposure	Epilepsy Subtypes/Diet	Level of Evidence
Kang et al. 2024 [24]	Immunology	HC = 29; IE = 49	Serum	↑ C3 and C4	Treated: n = 19 untreated: n = 30	Idiopathic TIER 2 Diet not standardised	Prognosis Study: II Retrospective Study
de la Fuente et al. 2012 [25]	Immunology	HC = 7; CE = 15; SID = 7; SRMA = 11; INF = 37; BT = 38; NON-INF = 40	CSF; Blood	No pleocytosis or protein elevation; bloodand CSF CRP and D-dimers not significantly different	Unknown	Idiopathic TIER 2 Diet not standardised	Prognosis Study: III Case-control study
Kostic et al. 2019 [26]	Inflammation	HC = 6; CE = 30; SE = 21	CSF; Serum	↑ serum IL-1β	Unknown	Idiopathic TIER 2 Diet not standardised	Prognosis Study: III Case-control study
Despa et al. 2024 [27]	Inflammation	HC = 8; CE = 29; SE = 10; RE = 10	Blood	↑ NLR	Variable	Idiopathic TIER > 1 Diet not standardized	Prognosis Study: II Retrospective Study
Koo et al. 2020 [28]	Inflammation	HC = 26; CE = 14; epileptic SE (MUO, BT) = 14; non epileptic SE (MUO, BT) = 12	Serum	↑ HMGB1; higher in >3 vs. ≤3 months	Treated with only ASM (phenobarbital, potassium bromide, or zonisamide)	Idiopathic TIER > 1 Diet not standardized	Prognosis Study: III Case-control study
von Rüden et al. 2020 [29]	Inflammation	HC = 28; CE = 9; SE = 12	Brain tissue	↑ TLR4 expression; ↑ HMGB1; ↑ HSP70 (piriform lobe)	Unknown	Convulsive seizures (focal and generalized) Diet not standardized	Prognosis Study: III Case-control study
Segers et al. 2017 [30]	Inflammation	HC = 28; CE = 38	Serum	No consistent ↑ CRP	Treated	Idiopathic TIER 2 Diet not standardized	Prognosis Study: III Case-control study
Paltrinieri et al. 2017 [31]	Inflammation	HC = 10; CE = 14; dogs with other CNS disorders = 20	Serum	↑ total CK and CK-BB activity ↓ Macro-CK2 activity	Unknown	Diet not standardised	Prognosis Study: II Retrospective Study
Merbl et al. 2014 [32]	Inflammation	HC = 12; CE = 7, SE (MUO, BT) = 10	CSF; Serum	↑ TNF-α and ↑ IL-6	Unknown	Idiopathic TIER 2 Diet not standardized	Prognosis Study: III Case-control study
Hemmeter et al. 2023 [33]	Immunology	HC = 57; IE/dyskinesia = 58	Serum, CSF	No specific human or murine neural autoantibodies detected	Unknown	Idiopathic subtype: unknown cause. Diet not standardised	Prognosis Study: III Case-control study
Knebel et al. 2022 [34]	Immunology	HC = 10; IE = 57	CSF; Serum (IL-17) and whole blood (Th17)	↑ Th17 cells and ↑ IL-17	Variable	Idiopathic Diet not standardised	Prognosis Study: III Case-control study
Baka et al. 2021 [35]	Proteome	HC = 9; CE = 17; SE = 7	CSF	Immune/ECM pathway alterations Differential expression of protein markers	9 treated 8 untreated	Idiopathic TIER > 1 Diet not standardized	Prognosis Study: III Case-control study
Phochantachinda et al. 2023 [36]	Proteome	HC = 4; drug resistance CE = 4	Plasma	Altered amyloid/inflammatory proteins	Treated	TIER > 1 Diet not standardized	Prognosis Study: III Case-control study
Baka et al. 2024 [37]	Proteome	HC = 9; CE untreated = 8 CE treated= 9 SE = 8	Serum	Differential expression of protein markers	Variable	Idiopathic TIER > 1 Diet not standardized	Prognosis Study: III Case-control study
Verdoodt et al. 2025 [38]	AA, inflam, OS, vit	HC = 39; CE = 49	Plasma	Oxidative stress signature	Treated with ASM	Idiopathic TIER 2 Diet standardized	Prognosis Study: III Case-control study
Hasegawa et al. 2014 [39]	AA	HC = 18; CE = 16; SE = 19	CSF	Altered amino-acid profile (e.g., ↑ glutamic acid)	Unknown	Idiopathic TIER 2 Diet not standardized	Prognosis Study: II Retrospective Study
Weber et al. 2012 [40]	Energy	HC = 7, CE = 101, SRMA = 95, CNS neoplasia = 39, IVDD = 61, MUO = 19, BM = 6	CSF, blood	↑ CSF:serum glucose ratio & ↓ CSF protein, cell count vs. SRMA	Unknown	Diet not standardised	Prognosis Study: III Case-control study
Verdoodt et al. 2025 [41]	MGBA	HC = 39; CE = 49	Feces	Dysbiosis; altered SCFA pathways	Treated with ASM	Idiopathic TIER 2 Diet standardized	Prognosis Study: III Case-control study
García-Belenguer et al. 2021 [42]	MGBA	HC = 12; CE before treatment = 10; CE aftter treatment = 9	Feces	↓ GABA- and SCFA-producing bacteria ASM no impact	Single treatment with phenobarbital or imepitoin >30 days	Idiopathic TIER = 1 Diet standardized	Prognosis Study: III Case-control study
Muñana et al. 2020 [43]	MGBA	HC = 13; CE = 13	Feces	No major Lactobacillus differences	Not treated	Idiopathic TIER > 1 Diet standardized	Prognosis Study: III Case-control study
Silvestrino et al. 2025 [44]	MGBA	HC = 17; CE = 19	Feces	↓ Microbial diversity; ↑ pro-inflammatory taxa	Unknown	Idiopathic TIER > 1 Diet not standardized but monitored	Prognosis Study: III Case-control study
Yonezawa et al. 2024 [45]	Lipid & OS	HC = 9; CE = 11; MUO = 12	CSF; Plasma	Dysregulated lipid metabolites	7 CE treated with ASM 1 CE under prednisolone	Idiopathic TIER 2 Diet not standardized	Prognosis Study: II Retrospective Study
Kluger et al. 2008 [46]	Lipid & OS	HC = 57; CE = 57	Serum	↑ Triglycerides (Tx CE)	28 CE under phenobarbital 29 CE phenobarbital and bromide	Idiopathic TIER 2 Diet not standardized but monitored	Prognosis Study: III Case-control study
Radaković et al. 2023 [47]	Lipid & OS	HC = 15; CE = 15	Blood	Systemic oxidative stress	Untreated	TIER > 1 Diet not standardized	Prognosis Study: III Case-control study
Rosendahl et al. 2023 [48]	Minerals & vit	HC = 19; CE = 19	Blood	↑ Cu, Se, Cr	18 treated	Idiopathic TIER > 1 Diet not standardized but monitored	Prognosis Study: III Case-control study
Rosendahl et al. 2023 [49]	Minerals & vit	HC = 42; CE = 63	Hair	Mineral imbalances	treated (n = 53); untreated (n = 10)	Idiopathic TIER > 1 Diet not standardized but monitor	Prognosis Study: III Case-control study
Vitale et al. 2019 [50]	Minerals & vit	HC = 50; CE = 92	Serum	Altered Se, Cu	Controlled CE (12), uncontrolled CE (42), and untreated CE (13)	Idiopathic TIER > 1 Diet not standardized but monitor	Prognosis Study: III Case-control study
Schmidt et al. 2022 [51]	Neurotransmitters	HC = 127; CE = 63	Urine	Distinct neurotransmitter profile (e.g., glycine, serotonin, norepinephrine/epinephrine ratio)	Treated with ASM (e.g.,phenobarbital, potassium bromide) and other variable therapy	Idiopathic TIER I (n = 15) TIER II (n = 48) Diet not standardized	Prognosis Study: III Case-control study
Ellenberger et al. 2004 [52]	Neurotransmitters	HC = 20; CE = 94; GE = 35	CSF	↓ GABA and ↓ Aspartate	Variable 42 CE treated with phenobarbital	Idiopathic TIER > 1 Diet not standardized	Prognosis Study: II Retrospective Study
Morita et al. 2005 [53]	Neurotransmitters	HC = 3; IE = 4 + 4	Blood	↑ extracellular Glu and Asp levels (during epileptiform activity) ↓ GLT-1 expression (cortex, thalamus) -perineuronal Glu accumulation (cortex)	Unknown	Idiopathic, subtype: genetic cause Diet not standardised	Prognosis Study: III Case-control study
Gesell et al. 2013 [54]	Endocannabinoid	HC = 16; CE = 40	CSF	↑ AEA, 2-AG	Unknown	Idiopathic Diet not standardized	Prognosis Study: II Retrospective Study
Kostic et al. 2023 [55]	Endocannabinoid	HC = 7; CE = 5; SE = 7	Brain tissue	↓ CB1R in CE dogs hippocampus vs. HC and SE	Unknown	Idiopathic, subtype: unknown cause. With cluster before euthanasia. Diet not standardised	Prognosis Study: II Retrospective Study
García-Gracia et al. 2024 [56]	miRNA	HC = 8; CE = 15 (drug-sensitive = 9; drug-resisance = 6)	Plasma	Distinct miRNA profile	Treated	TIER > 1 Diet not standardized	Prognosis Study: III Case-control study

Summary of the 33 original research studies identified through the PRISMA-based literature search, each comparing metabolic features of dogs with idiopathic epilepsy to healthy controls across cerebrospinal fluid, blood, brain tissue, or fecal samples. **Abbreviations:** 2-AG = 2-arachidonoylglycerol; AA = amino acids; AEA = anandamide; BM = bacterial meningoencephalomyelitis; BT= brain tumor; C3/4 = complement factor 3/4; CB1R = cannabinoid 1 receptor; CK (-BB) = creatine kinase–(brain isoform); CNS = central nervous system; CRP = C-reactive protein; CSF = cerebrospinal fluid; Ctl = controlled; ECM = extracellular matrix; HC = healthy controls; HMGB1 = high mobility group box 1; CE = canine idiopathic epilepsy; GE= genetic idiopathic epilepsy; IVDD = intervertebral disc disease; MGBA = microbiota–gut–brain axis; miRNA = micro-RNA; MUO = meningoencephalomyelitis of unknown origin; NLR = neutrophil-to-lymphocyte ratio; OS = oxidative stress; SE = structural epilepsy; SCFA = short chain fatty acids; SRMA = steroid-responsive meningitis-arteritis; TLR4 = Toll-like receptor 4; Tx = treated; vit = vitamins.

**Table 2:** Summary of metabolic system and pathway alterations in canine idiopathic epilepsy in comparison with human epilepsy.

Metabolic System/Pathway	Similarities Canine—Human	Divergence Canine—Human	Research Gaps or Limitations
**Acute phase response**	↑ CRP, neutrophil-to-lymphocyte ratio, CSF TNF-α, IL-1β; BBB dysfunction; ↑ xanthurenic acid	—	CRP variability across subtypes and age groups; specific plasma lipid alterations; ASM influence unclear
**DAMPs and tissue damage markers**	↑ Serum HMGB1 and CNS HSP70	↑ CK-BB CE, but not PWE	CK could not differentiate CE from other CNS diseases
**Innate immune system**	Complement involvement in both species	Complement cascade: ↑ C3/C4 in CE vs. ↓ C3/C4 in PWE	Functional impact on epileptogenesis unclear
**Adaptive immune system**	Th17 cells involved and ↑ IL-17	—	Role of coagulation markers (D-dimer); Importance of autoimmune encephalitis unclear; ASM influence unclear
**MGBA**	Tryptophan–kynurenine pathway alterations; ↓ fecal *Phascolarctobacterium*	Phylogeny of some microbial alterations differs	Peripheral histamine role in humans; alterations of *Prevotella* and *Escherichia-Shigella* in both directions for PWE; variability due to 16S rRNA sequencing
**Oxidative stress**	↑ AOPP	Lipid peroxidation inconsistent; selenium status opposite	ASM influence on oxidative markers unclear; driver for epileptogenesis or consequence?
**Lipid metabolism**	Therapeutic use of MCT diets	Specific lipid metabolites (OEA, 11,12-DHET) detected in dogs, not humans	ASM influence on lipid profile unclear
**Amino acid metabolism**	↑ Blood leucine; lysine acetylation parallels	Blood threonine: ↑ CE vs. ↓ PWE	Influence of timing post-seizure and ASM unclear
**Protein metabolism**	↑ Amyloid-β	Proteomic patterns differ (e.g., ↓ MMP-2 in CE)	Functional role of haptoglobin unclear
**Vitamins**	↓ Vitamin B6	ASM impact on vitamin B6 more documented in PWE	Optimal dosing and safety margins; ASM influence unclear
**Minerals**	↑ Cu/Zn ratio	Selenium: ↑ CE vs. ↓ PWE	Clinical relevance of manganese and chromium unclear
**Neurotransmission**	Serotonin involvement; GABA/glutamate ratio altered	Urinary NE/E ratio changes in CE; ↑ Plasma and CSF NE in PWE	Correlation with comorbidities unclear
**Endocannabinoid system**	Therapeutic use of CBD	CSF anandamide: ↑ CE vs. ↓ PWE Localisation CB1R in the brain	CB1 receptor modulation complexity
**Post-transcriptional regulation**	↑ miR-134 and miR-129 in drug-resistant cases	Limited canine data, mainly pilot studies	Inconsistent alterations for miR-223; different subtypes tested in CE vs. PWE

**Abbreviations**: 11,12-DHET = 11,12-dihydroxyeicosatrienoic acid; AOPP = advanced oxidation protein products; ASM = antiseizure medication; BBB = blood-brain barrier; C3/4 = complement factor 3/4; CB = cannabinoid; CBD = cannabidiol; CE = canine idiopathic epilepsy; CK-BB = creating kinase-brain isoform; CNS = central nervous system; CRP = C-reactive protein; CSF = cerebrospinal fluid; DAMPs = damage associated molecular patterns; E = epinephrine; HMGB1 = high mobility group box 1; HSP70 = heat shock protein 70; MCT = medium chain triglycerides; miR = microRNA; MMP-2 = matrix metallopeptidase 2; NE = norepinephrine; OEA = oleoylethanolamide; PWE = people with epilepsy; TNF = tumor necrosis factor.

## Data Availability

The data that support the findings from this review are available in the referenced publications. No new data was generated. The review protocol has not been registered.

## Supplementary Materials

The following supporting information can be downloaded at: https://www.mdpi.com/article/10.3390/nu18111734/s1, File S1: Preferred Reporting Items for Systematic reviews and Meta-Analyses extension for Scoping Reviews (PRISMA-ScR) Checklist; File S2: Literature Search.
